# Substrate Stiffness Mediates Formation of Novel Cytoskeletal Structures in Fibroblasts during Cell–Microspheres Interaction

**DOI:** 10.3390/ijms22020960

**Published:** 2021-01-19

**Authors:** Olga Adamczyk, Zbigniew Baster, Maksymilian Szczypior, Zenon Rajfur

**Affiliations:** 1 Institute of Physics, Faculty of Physics, Astronomy and Applied Computer Science, Jagiellonian University, 30-348 Kraków, Poland; olga.adamczyk@doctoral.uj.edu.pl (O.A.); zbigniew.baster@doctoral.uj.edu.pl (Z.B.); maksymilian.szczypior@student.uj.edu.pl (M.S.); 2 Jagiellonian Center of Biomedical Imaging, Jagiellonian University, 30-348 Kraków, Poland

**Keywords:** cytoskeleton, elastic substrates, endocytosis, latex microspheres, microtubules, actin, vimentin

## Abstract

It is well known that living cells interact mechanically with their microenvironment. Many basic cell functions, like migration, proliferation, gene expression, and differentiation, are influenced by external forces exerted on the cell. That is why it is extremely important to study how mechanical properties of the culture substrate influence the cellular molecular regulatory pathways. Optical microscopy is one of the most common experimental method used to visualize and study cellular processes. Confocal microscopy allows to observe changes in the 3D organization of the cytoskeleton in response to a precise mechanical stimulus applied with, for example, a bead trapped with optical tweezers. Optical tweezers-based method (OT) is a microrheological technique which employs a focused laser beam and polystyrene or latex beads to study mechanical properties of biological systems. Latex beads, functionalized with a specific protein, can interact with proteins located on the surface of the cellular membrane. Such interaction can significantly affect the cell’s behavior. In this work, we demonstrate that beads alone, placed on the cell surface, significantly change the architecture of actin, microtubule, and intermediate filaments. We also show that the observed molecular response to such stimulus depends on the duration of the cell–bead interaction. Application of cytoskeletal drugs: cytochalasin D, jasplakinolide, and docetaxel, abrogates remodeling effects of the cytoskeleton. More important, when cells are plated on elastic substrates, which mimic the mechanical properties of physiological cellular environment, we observe formation of novel, “cup-like” structures formed by the microtubule cytoskeleton upon interaction with latex beads. These results provide new insights into the function of the microtubule cytoskeleton. Based on these results, we conclude that rigidity of the substrate significantly affects the cellular processes related to every component of the cytoskeleton, especially their architecture.

## 1. Introduction

Micrometer-size beads are commonly used in biomedical studies. One area, where such microspheres are extensively used, is to study the process of phagocytosis where they serve as a model for cellular phagocytosis [1]. Moreover, latex beads are also employed in cellular mechanobiology studies, where they are usually used with optical or magnetic tweezers, that allow to manipulate the beads. These techniques can be applied to stimulate cells mechanically [2] as well as to measure various parameters of cells, such as cytoskeleton elasticity [3] or membrane viscoelasticity [4].

One type of a commonly performed experiment is the tether extraction [5,6,7]. It was described in 1989 by Ashkin and Dziedzic [8]. The experiment bases on attaching a bead to the outer membrane of a cell and then pulling a thin, membrane-derived thread out of it [9]. Primarily, this technique was used to estimate cell membrane properties such as its viscosity or the actin cytoskeleton–cell membrane interaction [5,9]. Currently, optical tweezers-pulled tethers are also used to study nanotubular protrusions like tubular nanotubes or filopodia [10,11].

Microspheres are frequently used to measure cell viscoelasticity. Unfortunately, measuring viscoelastic properties of cells is challenging and the results usually are burdened with high measurement errors. To perform this kind of experiment, usually, a trapped diamagnetic bead and dynamically perturbed by the laser beam is employed [12].

The main problem with applying bead trapping techniques to cells, tissues, and other biological samples is the need to use of a high-power laser beam which is harmful to these samples [13]. This is why cellular research is mostly performed using a near-infrared light (e.g., λ = 1 064 nm) [13], which is less harmful than a visible light [13,14]. There are also other aspects that may interfere with experiment’s results, including the interaction between beads and cell structures is often overlooked while planning an experiment.

As it was mentioned earlier, one of the applications of the beads trapping techniques is to measure elastic parameters of cells, which can be estimated by using polystyrene or latex beads [15]. It is obvious, that the beads should be completely biocompatible, i.e., they should not be toxic nor interfere with cellular structures.

The cytoskeleton is responsible mainly for elasticity and other mechanical properties of cells [16]. It consists of three components: microfilaments, intermediate filaments, and microtubules. There are many diverse functions of the cytoskeleton [16,17], however, from the point of cell elasticity, the most important function of the cytoskeleton is the transduction of mechanical signals [16]. The cytoskeleton is an important component of any cell, thus, for researchers working with cells, it is extremely important to closely investigate its behavior. The cytoskeleton is responsible for many important cellular processes essential for cell functioning. Yet, there are many cytoskeleton-mediated molecular mechanisms to be discovered. Without the cytoskeleton, basic processes such as cell migration, cell division or intracellular transport would not be possible. Furthermore, mechanical functions such as shaping the cell, mechanical strengthening, and keeping the cell organelles in proper position are equally significant. This is extremely important for animal cells as they do not have a rigid cell wall. The multitude and wide range of functions of the cytoskeleton make it an essential and interesting component of the cell.

Latex beads are widely used not only in biomechanical studies but also in different types of endocytosis experiments [18,19]. Molecular pathways of endocytosis and mechanosensing are often intercrossed, like in integrin-mediated adhesion or phagocytosis [20,21]. Integrins are the key proteins in the cell adhesion process. They are cell surface receptors of extracellular matrix (ECM) proteins, like integrin α5β1, a receptor for fibronectin [22,23]. Integrin binding to the ECM initiates formation of adhesion complexes and remodeling of the actin cytoskeleton [24]. On the other hand, previous studies show that integrins also mediate formation of phagosomal compartments [21]. Furthermore, Grinnell discovered that whether cells undergo adhesion-migration or phagocytosis pathway depends directly on the size of the object that cells get in contact with [25], what further shows that these processes are tightly connected. In many experiments, beads are often coated with extracellular matrix protein, such as fibronectin, to increase their affinity towards cells. However, the interaction activates not only mechanosensing pathway, but also initiates endocytotic (including phagocytotic) response, especially in the case of beads of smaller sizes [25]. One of the main question is: how does elasticity of substrate influence this interaction? The problem described above has already been studied, but it was described only partially [26,27,28].

Due to the development of the field of mechanobiology it is crucial to design and thoroughly characterize experimental methods used to study new, emerging questions. The rationale of this work was to check whether the latex beads have any influence on cells, especially the cytoskeleton. Initial results indicated that the latex beads interaction with the cell is more complex than one could expected and that led us to examine the observed changes in the cytoskeleton architecture in 3D with confocal microscopy.

Here, we describe how beads, commonly used in mechanobiological studies, influence cells. We examined the changes in the architecture of actin, microtubules, and intermediate filaments in a time dependent manner. Modification of cytoskeleton’s architecture should have quite a significant impact on the experimental results and it should be taken under consideration at the planning stage of the experiment. The last goal of our work was to investigate how culture substrate elasticity alters the observed phenomena. During that work, we discovered that novel cup-like structures were formed by microtubule cytoskeleton, which is not known for its active involvement in endocytosis, only on elastic substrates.

## 2. Results

### 2.1. Cytoskeleton Remodeling Due to External Stimuli

**Figure 1:**
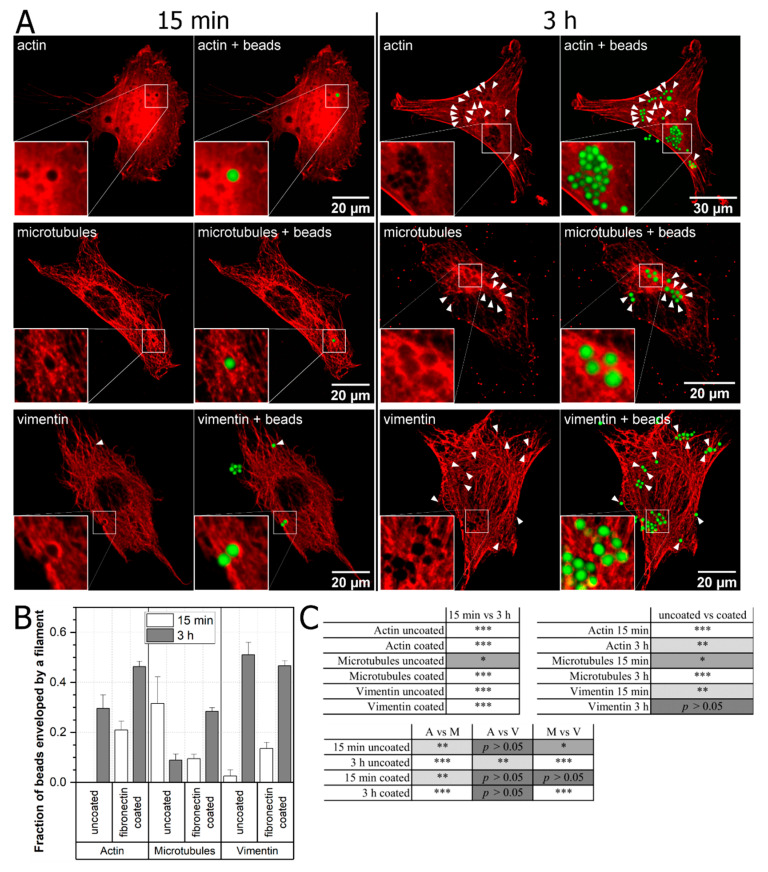
Latex beads cause the cytoskeleton rearrangement. MEF cells were plated on glass-bottom dishes and incubated with 2 µm diameter latex beads (coated with fibronectin and uncoated) for 15 min or 3 h, then fixed, stained for cytoskeletal components (separately) and visualized using confocal microscopy in 3D. Images are presented as sums of stacks. (**A**) Representative images of cytoskeletal structures in experiments with fibronectin-coated beads are shown, arrows point at spots where beads were enveloped by the cytoskeleton and visible changes could be observed. (**B**) Histogram showing the fraction of beads enveloped by a specific component of the cytoskeleton. Fully enveloped beads were counted and presented as a fraction of all beads interacting with cells. Data are presented as a percentage fraction ± SEM (actin, 15 min, uncoated, *n* = 16; actin, 3 h, uncoated, *n* = 71; actin, 15 min, coated, *n* = 129; actin, 3 h, coated, *n* = 523; microtubules, 15 min, uncoated, *n* = 19; microtubules, 3 h, uncoated, *n* = 135; microtubules, 15 min, coated, *n* = 285; microtubules, 3 h, coated, *n* = 905; vimentin, 15 min, uncoated, *n* = 39; vimentin, 3 h, uncoated, *n* = 100; vimentin, 15 min, coated, *n* = 192; vimentin, 3 h, coated, *n* = 581). (**C**) Two proportion Z-test was used to determine statistical significances between specific samples: (**upper left**) bead incubation duration: 15 min versus 3 h; (**upper right**) uncoated versus coated beads; (**lower left**) comparison between cytoskeletal components: (A)ctin, (M)icrotubules, and (V)imentin. * *p* < 0.05, ** *p* < 0.005, *** *p* < 0.001. Data are representative of at least two independent experiments.

To learn how cells respond to an external stimulus we analyzed cytoskeleton rearrangement caused by latex beads in time. We plated Mouse Embryonic Fibroblasts (MEF) 3T3 cells on glass-bottom dishes with a low confluence and cultured overnight. The next day we added 2 µm carboxylated fluorescent latex beads, uncoated or coated with fibronectin. Then, the cells were incubated with beads for 15 min or 3 h, and after that, fixed with 4% formaldehyde solution. After fixation, cells were stained against actin, α-tubulin, or vimentin and images of respective cytoskeleton components were captured using confocal microscopy. After 3D reconstruction, we noticed that the beads disturbed all types of cytoskeleton filaments and many of the beads were fully enveloped by the specific filament. The enveloped beads were counted and compared with the number of all the beads attached to the cells (Figure 1A, Supplementary Figure S1); fractions of enveloped beads by specific components of the cytoskeleton are shown in Figure 1B,C.

As expected, more fibronectin-coated than uncoated beads attached to cells in general, due to their higher affinity. Moreover, the general tendency was that the greater or at least the same fraction of coated beads were influencing the cytoskeleton rearrangement when compared to the uncoated ones. Furthermore, our results show that the bead–cytoskeleton interaction is time dependent. As expected, more beads were enveloped by the actin or the vimentin cytoskeleton after 3 h of interaction than after 15 min. However, in the case of microtubules, more uncoated beads were enveloped after 15 min than after 3 h of interaction (Figure 1B).

### 2.2. Influence of Cytoskeletal Toxins on Cell–Bead Interaction

**Figure 2:**
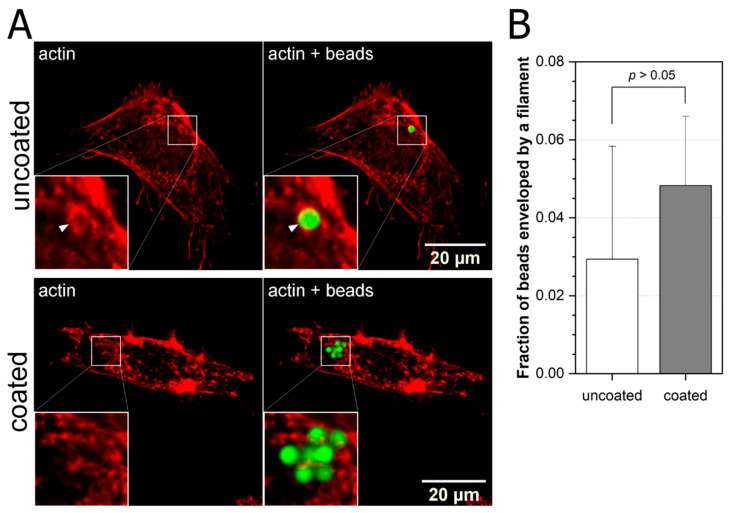
Lack of beads envelopment by actin filaments after treatment with cytochalasin D. (**A**) MEF cells were plated on glass-bottom dishes and cultured overnight then incubated with 2 µm latex beads (coated with fibronectin and uncoated) together with 20 µM cytochalasin D for 1 h and stained for actin. 3D images of cells were taken and analyzed. Representative images (as sum of stacks) are shown. (**B**) Beads enveloped by actin were counted and presented as fractions of all beads interacting with cells. Data are presented as a fraction ± SEM (uncoated, *n* = 34; coated, *n* = 145). Z-test was performed. Data are representative of at least two independent experiments.

To investigate how cytoskeleton targeting drugs influence the cytoskeleton–bead interaction, we treated MEF 3T3 cells with cytochalasin D (actin polymerization inhibitor), jasplakinolide (actin depolymerization inhibitor), or docetaxel (microtubules stabilizer). Cells were plated as described above and incubated with carboxylated fluorescent latex beads (2 µm diameter), uncoated or coated with fibronectin, together with 0.1% cytochalasin D for one hour. Cells were then fixed using 4% formaldehyde solution, stained against actin, and the cytoskeleton was visualized by confocal microscopy. Obtained 3D images were analyzed to count the beads attached to the cellular membrane or interacting with specific filament (Figure 2).

To study the influence of jasplakinolide and docetaxel, cells were plated as described above, after cells adhered to the surface we added jasplakinolide or docetaxel to media (final concentration 100 nM of either drug) and cultured overnight. On the next day, we added carboxylated YG fluorescent latex beads (2 µm diameter), uncoated or coated with fibronectin, incubated for 1 h with cells, then fixed samples with 4% formaldehyde solution, and stained against actin or α-tubulin, respectively. Such drug treatment totally inhibited previously observed cytoskeletal rearrangement.

### 2.3. Role of Substrate Elasticity in Mechanical Stimulus-Mediated Cytoskeleton Remodeling

**Figure 3:**
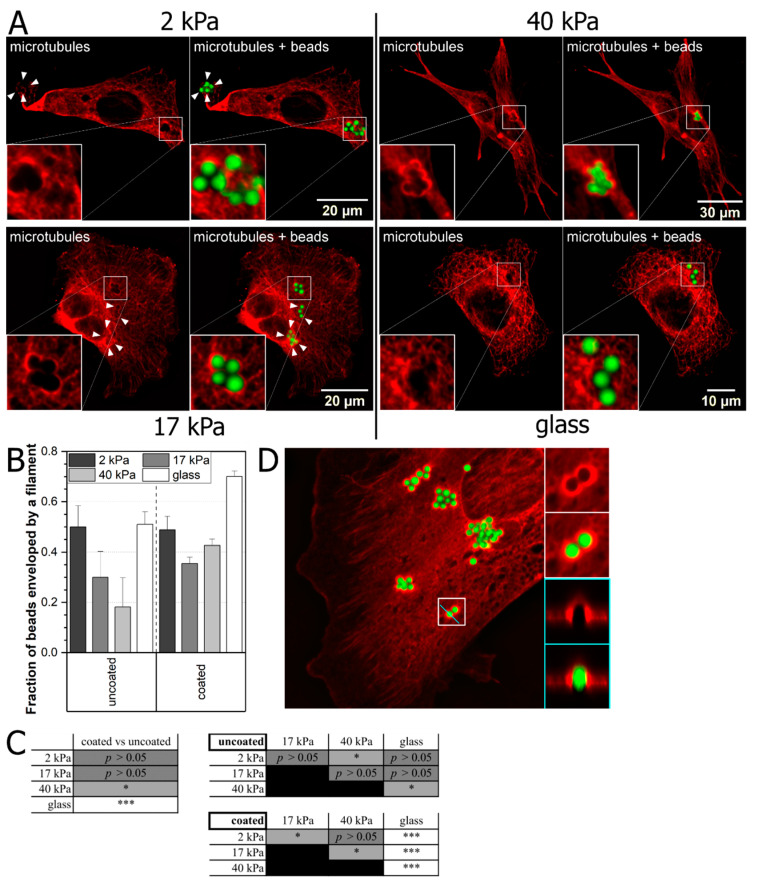
Influence of substrate elasticity on the latex bead-microtubule cytoskeleton interaction. (**A**) MEF cells were plated overnight on PA substrates of various elasticities or glass and incubated with 2 µm latex beads (coated with fibronectin and uncoated) for 1 h, then fixed, stained for the microtubule cytoskeleton and imaged using confocal microscopy in 3D. Images are presented as sums of stacks. Arrows point at spots where beads were enveloped by microtubule filaments and visible changes could be observed. Representative images of examined elasticities for experiments with fibronectin-coated beads are shown. (**B**) Microtubules enveloped beads were counted and presented as fractions of all beads interacting with cells. Data are presented as a fraction ± SEM (2 kPa, uncoated, *n* = 36; 2 kPa, coated, *n* = 88; 17 kPa, uncoated, *n* = 20; 17 kPa, coated, *n* = 369; 40 kPa, uncoated, *n* = 11; 40 kPa, coated, *n* = 394; glass, uncoated, *n* = 100; glass, coated, *n* = 434). (**C**) Two proportion Z-test was used to determine statistical significance between specific samples: (**left**) uncoated versus coated beads; comparison between different substrate elasticities using uncoated (**upper right**) and coated beads (**lower right**). * *p* < 0.05, *** *p* < 0.001. (**D**) MEF cell plated on substrate of 17 kPa of elasticity, scanned as a 3D image and showed as a sum of stack. Green color represents beads, red—microtubules. On the right side an enlarged section (white frame) and an axial projection of the 3D image (cyan frame) are presented. Data are representative of at least two independent experiments.

Nowadays, many cell studies are carried out on substrates which mimic mechanical characteristics of cells’ natural environment as close as possible, for example their elasticity/stiffness. To elucidate how substrate elasticity influence the cell–bead interaction we prepared polyacrylamide (PA) elastic substrates of three different elasticities—2 kPa, 17 kPa, 40 kPa. We plated MEF 3T3 cell as described before and cultured overnight on those substrates. Cells were then incubated with carboxylated YG fluorescent latex beads (2 µm diameter), uncoated (Supplementary Figure S2) or coated with fibronectin (Figure 3A), for one hour, then fixed, stained against α-tubulin, imaged, and analyzed as described above. Obtained data were compared with respective data for cells plated on glass substrate (Figure 3A–C).

**Figure 4:**
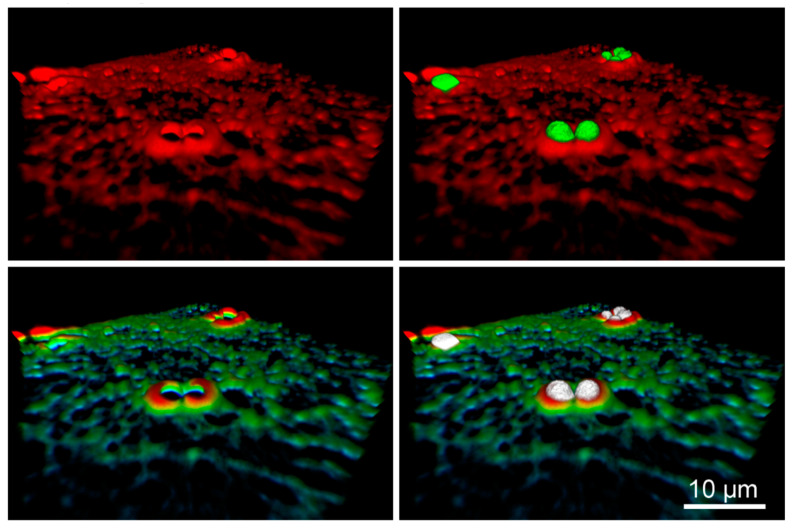
The microtubule cup-like structure in 3D rendering. The part of the same cell as in Figure 3D is shown. (**upper panels**) A 3D visualization of microtubule surface (red) surrounding beads (green). (**lower panels**) The height-color-coding for the same fragment for microtubule filaments (blue → green → yellow → red: low → high). Beads are shown in white. Images were rendered using FluoRender; rendering parameters in Supplementary Note S1.

Our results show that more coated beads are enveloped in comparison to uncoated ones on stiffer substrates (40 kPa and glass). In the case of cells plated on soft substrates (2 kPa and 17 kPa) we observed that bead coating has no influence on fractions of enveloped beads. What is noteworthy, on elastic substrates with Young modulus of 17 kPa and 40 kPa we observed formation of novel microtubules structure which resemble cups in places where the beads were located (Figure 3D). The formation of such cup-like structures in the case of actin filaments (Supplementary Figure S3), which take active part in the endocytosis process, was already described by Jaumouillé et al. [29] where cup-like structures formed of actin filaments were described as closely connected to the phagocytosis mechanism [30]. We observe the formation of similar structures made of microtubules, which, as it is postulated thus far, do not take an active role in the process of endocytosis. This clearly indicates that the microtubule cytoskeleton plays an active role, not described till now, in the process of phagocytosis, which has not been reported yet. A representative visualization of a cup is shown in Figure 4. Interestingly, similar structures do not form when the fibroblast cells were plated on glass substrate.

## 3. Discussion

### 3.1. Cytoskeletal Rearrangement

We examined the effect of latex beads with diameter of 2 µm (nonfunctionalized and functionalized with fibronectin), usually employed in endocytosis studies and optical tweezers-based measurements, on the cytoskeleton. Our study shows that beads alone, interacting with cells, significantly change the architecture of the cytoskeleton in as little as 15 min. Analysis of images of the actin, the microtubule and the vimentin cytoskeleton in cells grown on glass substrate clearly demonstrates what appears to be a void of respective filaments in the place where the beads were present (Figure 1A). The difference in the shape of filaments surrounding the beads 15 min and 3 h after placing them on the cell surface can be observed: after 15 min the actin and the vimentin cytoskeleton show a sign of beads engulfment attempt which is demonstrated by risen edges of respective filaments, surrounding the bead. In some cases (~20%), actin formed what appeared to be, a cup-like structure around the bead (Supplementary Figure S3). As expected, this effect is weaker with uncoated beads (Supplementary Figure S1). A careful analysis of the orthogonal cross-sections of the 3D images reveals that in many cases beads are enveloped by the respective filaments (Figure 3D) and because of the weak fluorescence signal from the top of the engulfment they look like a void in the filaments. We calculated and compared the percentage of fully engulfed beads by each cytoskeleton component and the results are as follows:

1. more fibronectin coated beads are engulfed by the respective cytoskeleton after 3 h incubation, though the percentage varies between them from 45% in case of actin and vimentin to 28% for microtubules;
2. for uncoated beads, the results are mixed—the highest percentage of engulfed beads is for vimentin (~50%) followed by actin (~30%) and then microtubules (~10%);
3. in the case of uncoated beads and microtubules, we see that beads are more efficiently engulfed after 15 min. than after 3 h of incubation. This counter intuitive result may point to the different mechanisms of bead engulfment by microtubules in comparison to actin and vimentin.

To determine the efficiency of latex beads enveloping by every filament type, we compare fractions presented in Figure 1B (after 15 min and 3 h of bead–cell interaction). We observe that in the case of actin, the very small percentage of uncoated (UC) beads is engulfed by actin, while 20% of fibronectin coated (FC) beads are enveloped after 15 min. After 3 h, we can see that almost 30% of UC beads and around 45% of FC beads are fully enveloped. In case of vimentin, the process of bead envelopment has different dynamics: 5% of UC and 15% of FC beads are enveloped by vimentin but after 3 h more UC beads (50%) than FC beads (45%) are enveloped by vimentin. When we look at microtubules, the picture is different than in previous two cases: surprisingly, after 15 min almost 30% of UC beads and only 8% of FC are enveloped by microtubules. Then, after 3 h of incubation, the percentage of enveloped UC beads drops to 8% but of enveloped FC beads increases to 27%. This shows that each cytoskeleton component interacts differently with UC and FC beads. Our data also show that the cytoskeleton’s role in the process of endocytosis is much more complex than it was thought thus far, especially when considering the time course of such process.

Summarizing this part, we can conclude, that the beads cause the fibroblast cytoskeleton’s rearrangement in as little as 15 min of interaction and many of the beads are fully engulfed by the cytoskeleton. Additionally, after 3 h beads are located closer to the nucleus (data not shown), what is in agreement with previous study [31]. One may speculate that actin treadmilling processes are responsible for this effect.

### 3.2. Cytoskeletal Toxins

The cytoskeleton rearrangement after latex beads settlement poses a question on how specific cytoskeletal inhibitors influence observed changes. To check this, we used SiR-Actin and SiR-Tubulin dyes containing cytoskeletal drugs jasplakinolide and docetaxel, respectively, to observe cytoskeletal changes in real time. Jasplakinolide inhibits the depolymerization of actin filaments [32] and docetaxel, which is a strong cytoskeletal drug used in chemotherapy, stabilizes microtubules [33]. Both of them have a major impact on the cytoskeleton and prevent the rearrangement of cytoskeletal components. After drug application, we did not observe any changes in the structure of actin or microtubules (data not shown). The beads settled on the dorsal side of cells and did not cause any visible changes in the cytoskeleton.

Following this lead, we checked the effect of cytochalasin D which prevents actin polymerization (opposite to jasplakinolide) [34]. In this case, we observed changes in the structure of actin filaments after cytochalasin application—it was difficult to see visible stress fibers and a very small percentage of beads were enveloped—5% fibronectin coated beads, 3% uncoated versus previously described 30–40%. This suggests that impaired regulation of cytoskeleton homeostasis lowers cells ability to respond to mechanical stimulus. It also shows that deformations caused by beads employed by optical tweezers-based methods have a significant impact on the architecture of a properly functioning cytoskeleton.

### 3.3. Elastic Polyacrylamide (PA) Substrates

Several previous studies demonstrated that cells behave differently when cultured on substrates with different elasticities [35,36]. To address this issue, we analyzed the architecture of the microtubule cytoskeleton, one of the components of the cytoskeleton, in fibroblasts plated on polyacrylamide elastic substrates with different elasticities—40 kPa, 17 kPa, and 2 kPa—and compared them to the ones plated on glass. As in previous experiments, we used two kinds of beads—uncoated and coated with fibronectin. After quantitative analysis of 3D images, we noticed an intriguing relationship. First, in the case of glass and 40 kPa substrate we observed a bigger percentage difference between envelopment of nonfunctionalized and fibronectin coated beads than in the case of softer substrates—17 kPa and 2 kPa (Figure 3B). This means, that on the softer substrates the beads are enveloped almost with the same efficiency for uncoated and coated ones. Second, even more striking, is that in the case of elastic substrates of 17 kPa and 40 kPa, we observed for the first time, the formation of specific, cup-like structures made of microtubules (Figure 4), which are not present in cells plated on glass. To our knowledge, this type of filament has not been reported to participate in phagocytosis directly, thus the effect was unanticipated. There are some indications that microtubules are able to create cup-like structure during phagocytosis [37], but this has not been directly observed yet. It also shows that mechanical parameters of the cellular microenvironment influence cellular regulatory processes.

Summarizing, our study shows, that latex beads change the architecture of the actin, the microtubule and the intermediate cytoskeleton of fibroblast cells. This effect is more pronounced when beads were functionalized with fibronectin. When cytoskeletal drugs, interfering with the function of the cytoskeleton, were used, its remodeling was almost unnoticeable. More importantly, we report for the first time, that

1. the effect of bead envelopment occurs, especially in the cell lamella,
2. the bead envelopment depends on the elasticity of the cellular substrates,
3. microtubules in cells cultured on soft cellular substrates, form cup-like structures surrounding beads placed on their dorsal surface.

These results can be extremely important in studies of the role and behavior of microtubules. According to current knowledge, microtubules do not participate in the process of endocytosis, but our results indicate that this may not be the case. Formation of the cup-like structures by microtubules on elastic substrates shows that these cytoskeletal elements may take part in the endocytosis process. This also implies that in future works, where polystyrene or latex beads are employed, for example phagocytosis or optical tweezers experiments, this effect has to be taken into account. The preservation of intact structures in living cells is sometimes of utmost interest and all factors which can have an impact on those structures have to be taken into consideration.

## 4. Materials and Methods

### 4.1. Cell Culture

We used MEF 3T3 Swiss mouse embryonic fibroblast cell line. Cells were cultured in DMEM Low Glucose medium (L0066, Biowest, Nuaillé, France) supplemented with 10% Gibco FBS (10270106, Thermo Fisher Scientific, Waltham, MA, USA), and 100 I.U./mL Penicillin and 100 μg/mL Streptomycin Solution (L0022, Biowest, Nuaillé, France) at 37 °C, 5% CO_2_, and 100% humidity. Prior to the experiment, cells were cultured overnight in a fresh medium and plated in a 35 mm glass bottom dishes with thickness #1 (0.13–0.16 mm) or #1.5 (0.16–0.19 mm) designed for high resolution imaging (Cellvis, Mountain View, CA, USA).

### 4.2. Beads and Bead Coating

Fluoresbrite YG Carboxylate Microspheres with diameter 2 µm were obtained from Polysciences (09847, Polysciences Inc., Warrington, PA, USA), are made of latex and can be visualized by fluorescent microscopy (441 nm absorption and 486 nm emission). Beads were coated with fibronectin from bovine plasma (341631, Merck, Darmstadt, Germany) in concentration of 0.7 mg/mL using PolyLink Protein Coupling Kit (24350-1, Polysciences Inc., Warrington, PA, USA), according to the manufacturers’ protocols. In brief, pellet of microparticles was suspended in PolyLink Coupling Buffer twice and the whole suspension was mixed with PolyLink EDAC solution. After these steps, fibronectin was added, incubated in room temperature with gentle mixing for 1 h, centrifuged twice, and resuspended in PolyLink Wash/Storage Buffer. The mixture was stored in 4 °C. The binding of the protein to the beads has been verified spectrophotometrically by measuring absorbance of suspension at 280 nm, according to the manufacture’s advice.

### 4.3. Cell Labeling and Immunoflurescence

Cells were fixed with 4% formaldehyde solutions (47608, Sigma-Aldrich, St. Louis, MO, USA) for 10 min, permeabilized for 7 min in 0.1% Triton X-100 (T8787, Sigma-Aldrich, St. Louis, MO, USA). In the cases of antibody staining, the non-specific sites were blocked with 3% BSA PBS buffer (A7906, Sigma-Aldrich, St. Louis, MO, USA) for 30 min, then stained for α-tubulin (1:500 in 3% BSA PBS buffer; T9026, Sigma-Aldrich, St. Louis, MO, USA) or vimentin (1:100 in 3% BSA PBS buffer; EPR3776, Abcam, Cambridge, UK) overnight. Then, Alexa Fluor 555-labeled goat anti-mouse IgG (H+L) antibody (1:100 in 3% BSA PBS buffer; A21422, Thermo Fisher Scientific, Waltham, MA, USA) or Alexa Fluor 633-labeled goat anti-rabbit IgG (H+L) antibody (1:50 in 3% BSA PBS buffer; A21070, Thermo Fisher Scientific, Waltham, MA, USA) for α-tubulin and vimentin, were added. For actin staining, cells were fixed, permeabilizated and incubated with DyLight 633-conjugated phalloidin (1:100 in PBS buffer; 21840, Thermo Fisher Scientific, Waltham, MA, USA) for 45 min.

### 4.4. Cytoskeletal Drugs

Cytoskeleton targeting drugs experiments were carried out with cytochalasin D solution (C8273, Sigma-Aldrich, St. Louis, MO, USA), which prevents polymerization of actin. Jasplakinolide (enhances polymerization of actin filaments) and docetaxel (stabilizes microtubules) were included in dyes Cytoskeleton Kit (SiR-Actin and SiR-Tubulin, 652 nm absorption, 674 nm emission) (CY-SC006, Cytoskeleton, Inc., Denver, CO, USA). The dyes were diluted to 100 nM of concentration. To inhibit actin polymerization, 20 µM cytochalasin D solution was applied for one hour. Experiments were made according to the manufacturers protocols and SiR drugs were incubated with cells overnight in an incubator.

### 4.5. Preparation of Polyacrylamide (PA) Gel Substrates with Different Mechanical Properties

PA gel substrates with different stiffnesses were fabricated based on standard procedures [38]. In brief, glass-bottom dishes were treated with a solution of 3-(Trimethoxysilyl)propyl methacrylate (440159, Sigma-Aldrich, St. Louis, MO, USA), 99.5% acetic acid, and 96% ethanol (1:1:14 ratio) for 30 min, washed twice with 96% ethanol, and dried. The proportions for preparation of the substrates with elasticity of 40 kPa, 16.7 kPa, and 2 kPa were taken from protocol [39]. Then, 13 µL of specific solution was placed at the center of a glass-bottom dish, covered with a glass coverslip, and incubated for 1 h in room temperature. After this time, the coverslip was removed, then the substrate was incubated with 2 µg/mL Sulfo-SANPAH (22589, Thermo Fisher Scientific, Waltham, MA, USA) under ultraviolet light for 5 min. Gels were rinsed with a sterile PBS solution and incubated with 10 µg/mL fibronectin at 4 °C overnight.

### 4.6. Confocal Microscopy and Data Analysis

Confocal images were acquired using Zeiss LSM 710 confocal module set on Zeiss Axio Observer.Z1 inverted microscope (Carl Zeiss Microscopy GmbH, Jena, Germany) using an oil immersion 40x 1.4 NA Plan-Apochromat objective. Before analysis, z-stack images were deconvolved using Batch Deconvolution Fiji v0.49 plugin (https://github.com/Mechanobiology-Lab/BatchDeconvolution) [40,41,42,43] with Ricardson-Lucy deconvolution algorithm [44,45]. PSF was calculated using Gibbson-Lanni PSF model [46] with the highest accuracy and parameters matching acquisition. All calculations were made using Fiji ImageJ environment [47,48]. 3D rendering was made using FluoRender 2.24 software (https://www.sci.utah.edu/software/fluorender.html) [49].

### 4.7. Statistical Analysis

Two proportion Z-test was used to analyze difference significance between the results.

## Data Availability

The data that support the findings of this study are available from the corresponding author upon reasonable request.

## Supplementary Materials

The following are available online at https://www.mdpi.com/1422-0067/22/2/960/s1.
