# Supplementary material for: Substrate Stiffness Mediates Formation of Novel Cytoskeletal Structures in Fibroblasts during Cell–Microspheres Interaction
Source: Int J Mol Sci. 2021 Jan 19;22(2):960. doi: 10.3390/ijms22020960 (PMC7835802; doi:10.3390/ijms22020960)
Supplement: Supplementary file 1 [file ijms-22-00960-s001.pdf]

Supplementary Materials for

Substrate Stiffness Mediates Formation of Novel Cytoskeletal Structures in Fibroblasts during Cell–Microspheres Interaction

Olga Adamczyk [1], Zbigniew Baster [1], Maksymilian Szczypior [1] and Zenon Rajfur [1,2,*]

[1] Institute of Physics, Faculty of Physics, Astronomy and Applied Computer Science, Jagiellonian University, 30-348 Kraków, Poland; olga.adamczyk@doctoral.uj.edu.pl (O.A.); zbigniew.baster@doctoral.uj.edu.pl (Z.B.); maksymilian.szczypior@student.uj.edu.pl (M.S.)
[2] Jagiellonian Center of Biomedical Imaging, Jagiellonian University, 30-348 Kraków, Poland
* Correspondence: zenon.rajfur@uj.edu.pl

This PDF file includes:

Supplementary Figures S1 to S3

Supplementary Note S1

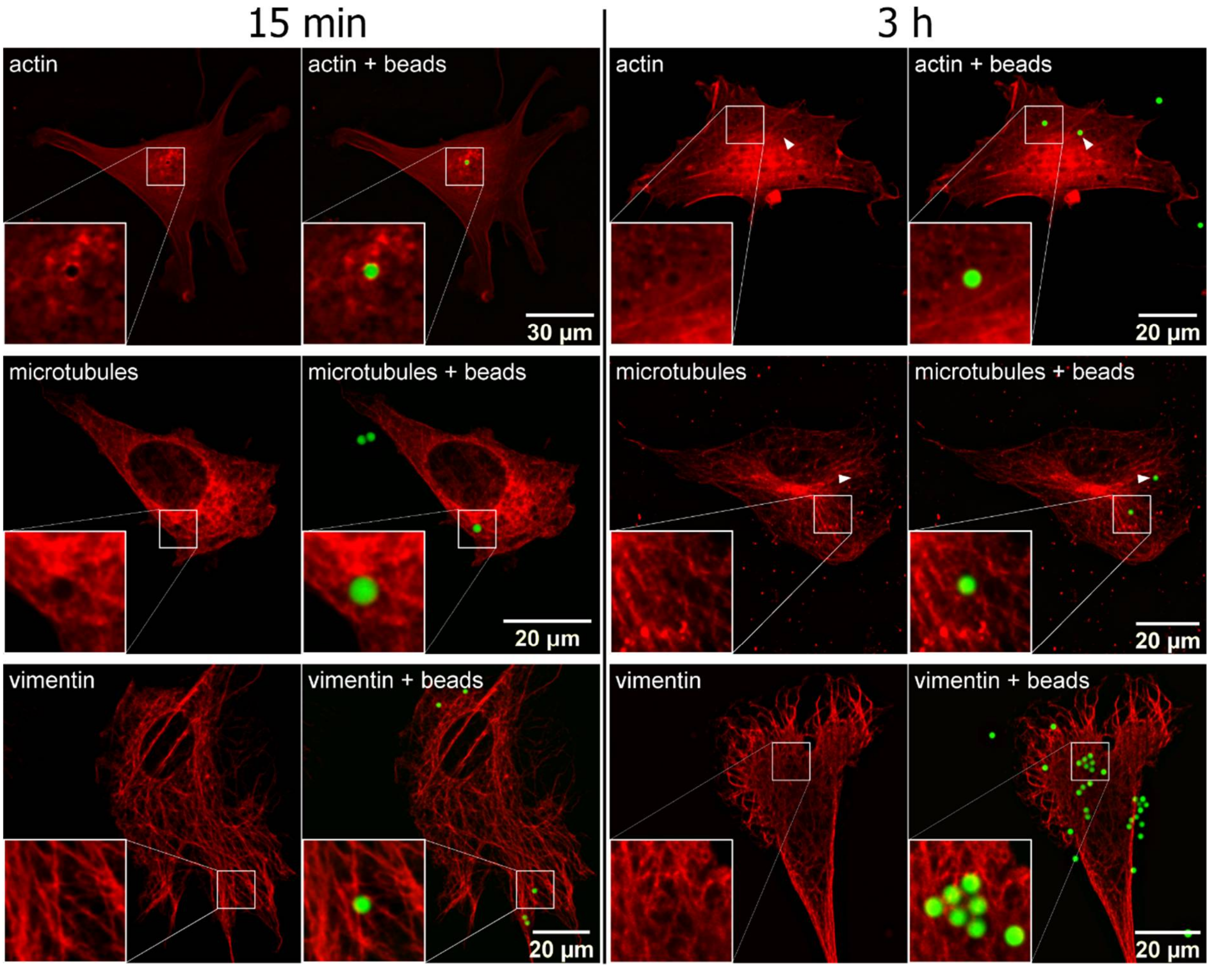

Supplementary Figure S1. Uncoated latex beads remodel cytoskeleton in time. MEF cells were plated on glass-bottom dishes and cultured with uncoated 2 µm latex beads for 15 min or 3 h, then fixed, stained for cytoskeletal components (separately) and imagined using confocal microscopy in 3D. Images presented as sum of stacks. Arrows point at spots where beads were enveloped by the cytoskeleton and visible changes could be observed. Representative images are shown

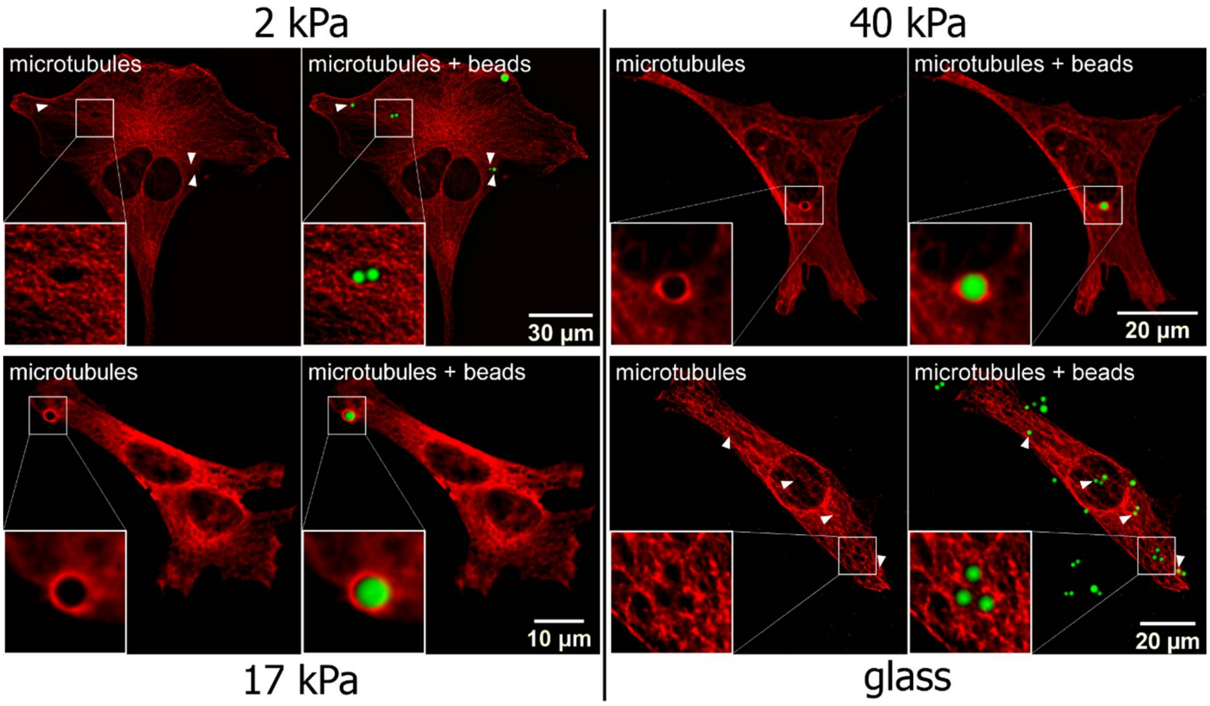

Supplementary Figure S2. Influence of substrate elasticity on latex bead-microtubule cytoskeleton interaction. MEF cells were plated overnight on PA substrates of various elasticities or glass and cultured with uncoated 2 µm latex beads for 1 h, then fixed, stained for microtubules cytoskeleton and imagined using confocal microscopy in 3D. Images are presented as sums of stacks. Arrows point at spots where beads were enveloped by the cytoskeleton and visible changes could be observed. Representative images for examined elasticities are shown.

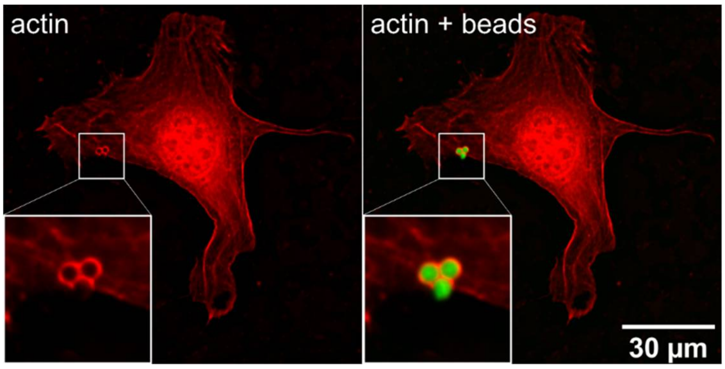

Supplementary Figure S3. A cup-like structure formed with actin filaments. MEF cells were plated overnight on glass-bottom dishes and cultured with coated 2 µm latex beads for 15 min, then fixed, stained for actin filaments and imagined using confocal microscopy in 3D. Images presented as sum of stack.

Supplementary Note S1. Parameters table from Figure 4 FluoRender rendering.

	Microtubules	*Beads*
Gamma	0.52	0.33
Saturation	4947	17953
Luminescenice	17953	17241
Alpha	17953	17953
Extract Boundary	0.0035	0.0555
Treshold	1973 / 17975	0/17953
Sample Rate	2.0	2.0
